# Comparison of the effect of modified intermittent fasting and daily calorie restriction on sleep quality, anthropometric data, and body composition in women with obesity or overweight: study protocol of a randomized controlled trial

**DOI:** 10.1186/s13063-023-07070-0

**Published:** 2023-01-16

**Authors:** Saeedeh Hosseini Hooshiar, Akram Yazdani, Sadegh Jafarnejad

**Affiliations:** 1 Research Center for Biochemistry and Nutrition in Metabolic Diseases, Kashan University of Medical Sciences, Kashan, Islamic Republic of Iran; 2 Department of Biostatistics and Epidemiology, Kashan University of Medical Sciences, Kashan, Islamic Republic of Iran

**Keywords:** Sleep quality, Intermittent fasting, Anthropometric indices, Pittsburgh Sleep Quality Index, Weight loss

## Abstract

**Background:**

Sleep disturbances are common in nearly one-third of adults. Both low quality of sleep and sleep time could be related to increased obesity. An increase in visceral adipose tissue can result in the secretion of inflammatory cytokines. Inflammatory cytokines can lead to a disturbance of the sleep-wake rhythm. Therefore, weight loss may improve sleep quality and duration. Intermittent fasting diet as a popular diet reduces body weight and improves anthropometric indices. This study is performed to further investigate the effect of a modified intermittent fasting diet on sleep quality and anthropometric indices.

**Methods:**

This is an open-label randomized controlled trial to evaluate the effect of daily calorie restriction (control) and modified intermittent fasting (intervention) on sleep quality, anthropometric data, and body composition in women with obesity or overweight for 8 weeks. Fifty-six participants will be classified using stratified randomization based on body mass index (BMI) and age. Then, participants will be assigned to one of the two groups of intervention or control using the random numbers table. The sleep quality, daytime sleepiness, and insomnia will be evaluated by using the Pittsburgh Sleep Quality Index (PSQI), the Epworth Sleepiness Scale (ESS), and the Insomnia Severity Index respectively. The primary outcomes chosen for the study were as follows: the difference in sleep quality, daytime sleepiness, insomnia, BMI, fat-free mass (FFM), body fat mass, waist circumference, and waist-to-hip ratio from baseline to 8 weeks. Secondary outcomes chosen for the study were as follows: the difference in hip circumference, the visceral fat area, percent body fat, soft lean mass, skeletal muscle mass, extracellular water ratio, and total body water from baseline to 8 weeks.

**Discussion:**

This study will investigate the effect of intermittent fasting intervention compared with daily calorie restriction on sleep quality and anthropometric indices. The information gained will enhance our understanding of fasting interventions, which can be used to improve clinical dietary recommendations. The findings will help to disclose as yet the unknown relationship between diet and sleep quality.

**Trial registration:**

Iranian Registry of Clinical Trials IRCT20220522054958N3. Registered on 8 July 2022. https://www.irct.ir/trial/64510.

## Background

Sleep disturbances affect a great number of the population. Nearly one-third of adults have one or more symptoms of sleep disturbances. Women and older people suffer more from insomnia than others. Some studies indicated that sleep quality is related to food intake [1]. Low sleep quality affects subjects with severe obesity and with moderate obesity [2, 3]. Inflammatory cytokines can lead to a disturbance of the sleep-wake rhythm [3]. Therefore, weight loss can improve sleep quality and duration [4]. Gangwisch et al. [5] in the National Health Examination Survey (NHANES) reported that participants with higher body mass index (BMI) had lower sleep duration than individuals with lower BMI.

Calorie restriction as first-line therapy has been considered for weight loss in obese patients [6]. Adherence to daily calorie restriction diets is low because the energy requirement is restricted every day [7]. Recently, intermittent fasting has emerged as a new diet for weight reduction. It increases metabolic health in addition to losing body weight [8]. Several studies demonstrated a 3 to 7% reduction in body weight under a fasting diet within 8 to 12 weeks [9]. What has remained unclear is whether intermittent fasting or calorie restriction will further improve anthropometric indices [6]. Other studies propose that alternate-day fasting may result in the preservation of fat-free mass and reduction of visceral adipose tissue in comparison with calorie restriction [10]. In a systematic review conducted by Trepanowski et al. [6], an alternate-day fasting diet and similar interventions reduced body weight similar to daily calorie restriction diets but reduced waist circumference greater than daily calorie restriction [6, 11]. They showed that both calorie restriction and alternate-day fasting increased the fat-free mass, and did not have an effect on the visceral adipose tissue [6]. Hutchison et al. [12] reported that intermittent fasting for 8 weeks resulted in greater fat mass and weight loss compared with calorie restriction in women with obesity and overweight. Previous studies indicated that calorie restriction improves sleep quality [13–15], but there are very limited studies on the effect of fasting on sleep quality. In a recent study by Teong et al. [16], no significant difference was seen between calorie restriction and intermittent fasting on sleep quality in women with obesity or overweight [16]. Therefore, this study will be performed to address this knowledge gap and further investigate the effect of a fasting diet on sleep and anthropometric indices.

## Methods/design

### Study design and participants

This is a randomized, controlled, parallel-group trial to compare the effect of a modified intermittent fasting diet and daily calorie restriction diet on sleep quality and anthropometric indices in women with obesity or overweight over 8 weeks. The framework of the trial is superiority. Participants will be selected from the Health Centers of Kashan University of Medical Sciences by simple random sampling. Participants will be randomly assigned into two groups, daily calorie restriction (control) and fasting (intervention). The protocol was approved by the Ethics Committee of Kashan University of Medical Sciences (IR.KAUMS.MEDNT.REC.1401.046) and was registered at the Iranian Registry of Clinical Trials (IRCT20220522054958N3). All participants will give written consent before any study-related procedure. Inclusion criteria include obese or overweight (40 >BMI ≥25) women between 18 and 50 years old. Exclusion criteria include breastfeeding; pregnancy; having chronic diseases such as heart disease, hypertension, diabetes, and gastrointestinal disorders; smoking; weight loss of 1 to 2 kg in the past month; alcohol abuse; following a specific diet or taking a specific medication; overnight shifts; taking dietary supplements; and having mental or psychological diseases. The obese or overweight (40 >BMI ≥25) women between 18 and 50 years old will be included in the study, provided they meet the inclusion criteria.

### Randomization

Fifty-six participants will be selected from the Health Centers of Kashan University of Medical Sciences by Simple Random Sampling according to the inclusion and exclusion criteria. A trained nutritionist will enroll participants. Eligible participants will be stratified by age and BMI to ensure between-group homogeneity. Participants per stratum will be assigned to one of the two groups “modified intermittent fasting” (intervention) and “daily calorie restriction” (control) after baseline investigations. Individuals cannot be blinded to allocation because of the nature of the intervention. An independent statistician will generate the allocation sequence by using random numbers’ table and the statistician will be blinded throughout the entire trial.

### Sample size calculation

We based our sample size calculation on fat-free mass (FFM) data of a previously published randomized controlled trial [17]. The mean and standard deviation of FFM were 63.4 and 3.4 (kg) in the intervention group and 66.9 and 4.9 (kg) in the control group. To detect a clinically significant difference in the outcome measures (sleep quality, daytime sleepiness, insomnia, BMI, FFM, body fat mass, waist circumference, waist-to-hip ratio, hip circumference, the visceral fat area, percent body fat, soft lean mass, skeletal muscle mass, extracellular water ratio, and total body water) with an alpha error of 5% and power of 80%, 23 women will be needed in each group. We presume a drop-out rate of 20%, based on clinical study experience, and thus 28 women will be recruited in each group.

$$n=\frac{{\left(Z1-\frac{\alpha }{2}+Z1-\beta \right)}^2\left({\sigma}_1^2+{\sigma}_2^2\right)}{\left({\mu}_1-{\mu}_2\right)}$$

### Study hypothesis

We hypothesize that individuals with obesity or overweight will have more sleep disorders and that modified intermittent fasting diet will most benefit this group.

## Outcome measures

### Primary outcomes

The primary outcomes of the study are the differences between the two groups with regard to the changes from baseline values in the following variables: Pittsburgh Sleep Quality Index (PSQI), Epworth Sleepiness Scale (ESS), Insomnia severity index, BMI, FFM, body fat mass, waist circumference, and the waist-to-hip ratio.

### Secondary outcomes

The secondary outcomes of the study are the differences between the two groups with regard to the changes from baseline values in the following variables: the hip circumference, the visceral fat area, the percent body fat, the soft lean mass, the skeletal muscle mass, the extracellular water ratio, and the total body water.

### Intervention

**Fig. 1:**
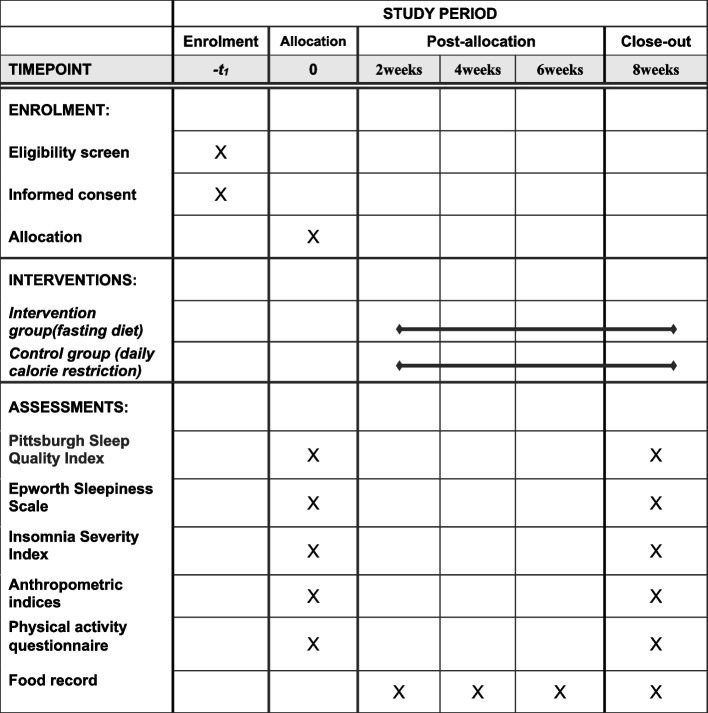
The schedule of enrolment, interventions, and assessments

**Fig. 2:**
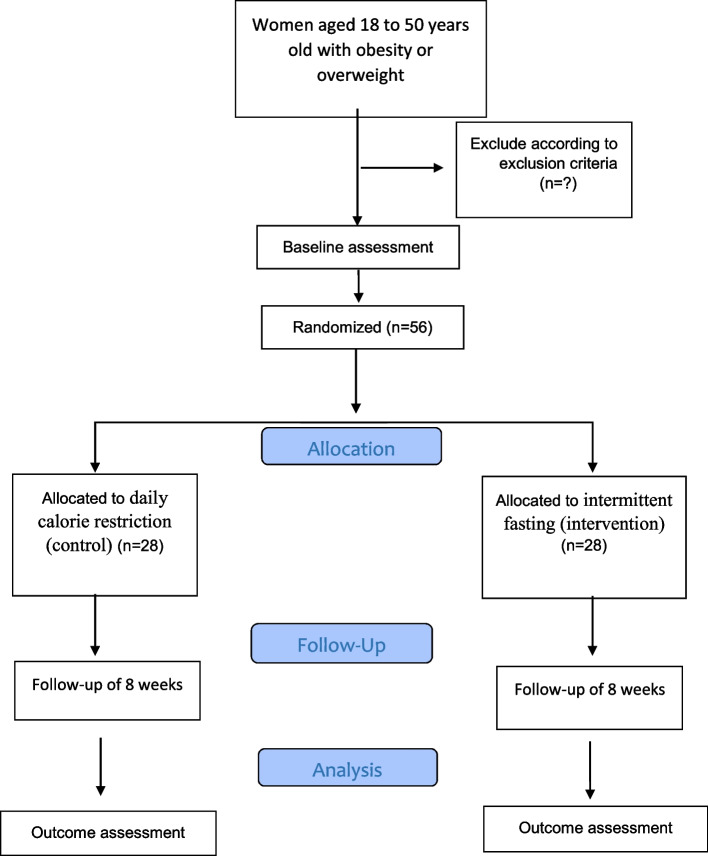
Flow chart of the intervention

The flow chart of the intervention is shown in Fig. 2. In this study, 56 participants will be selected from the Health Centers of Kashan University of Medical Sciences by Simple Random Sampling according to the inclusion and exclusion criteria. Eligible participants will be stratified by age and BMI to ensure between-group homogeneity. Participants per stratum will be assigned to one of the two groups “modified intermittent fasting” (intervention) and “daily calorie restriction” (control) after baseline investigations. All participants will be required to follow their diet which is prescribed for 8 weeks based on their group and daily energy requirements. The energy requirements for the individuals will be calculated using the Mifflin equation [18]. Intermittent fasting will involve alternating periods of feeding and fasting, on an every-other-day basis (subjects will consume only 25% of the recommended calorie intake on the fasting days and then will consume 100% of the calculated daily energy requirements on feeding days). At midnight each day, the fast and feed days will begin. All fast day meals will be consumed as lunch between 12.00 pm and 2.00 pm to ensure that each subject will be undergoing the same duration of fasting. Participants will be permitted to drink energy-free beverages (such as water, tea, and green tea), coffee without sugar (<400 mg caffeine daily), and non-starchy vegetables (such as lettuce, green leaf, cucumber, and tomato). They will be encouraged to consume plenty of water. On feeding days, the participants will be provided foods at 100% of the calculated daily energy requirements (three main meals and three snacks) and will be advised to eat breakfast at 8:00, lunch at 13:00, and dinner at 20:00. Additionally, participants will be advised to have the first snack at 10:00, the second snack at 16:00, and the last snack at 22:00. The control group daily will consume 63% of their energy requirement (three main meals and three snacks) and will be advised to eat breakfast at 8:00, lunch at 13:00, and dinner at 20:00. Also, the first snack should be eaten at 10:00, the second snack at 16:00, and the last snack at 22:00. Both groups will follow their diet for 8 weeks. Control and intervention group subjects must prepare all their meals at home. All subjects in the two groups will be asked to keep their regular physical activity throughout the study. Daily dietary carbohydrates, fat and protein will account for 55, 30, and 15% of energy requirement, respectively. All participants will have the same number of contacts with the dietician at the on-site visits and phone visits for questions. Implementation adherence will assess by a food record questionnaire. It will be completed by the participants once every 2 weeks, 3 days (2 days/week and 1-day weekend) a week. If the total caloric intake of patients will be between 80 and 110% of the prescribed, there will be adequate adherence [19]. The following will be prohibited during the study: taking a special diet, taking a special supplement or medicine. An overview of the study design, interventions, and assessments to be conducted during the study and their timing is presented in Fig. 1 (enrolment, interventions, and assessments) and Fig. 2 (flow chart of the intervention).

### Plans for participant retention and adherence to the diet

To control the adherence to the diet by the participants, once every 2 weeks, food record questionnaires will be completed 3 days (two normal days and one day off) a week. Researchers will make regular phone calls to participants to reinforce what they will be doing and to encourage them to continue. Also, participants will be monitored in weekly phone interviews. The data of subjects who discontinue or deviate from intervention protocols for any reason will be analyzed with intention-to-treat.

### Criteria for discontinuing or modifying allocated interventions

The criteria for discontinuing allocated interventions for a given trial participant include the following: calorie intake below 80% or above 110% of their recommended calorie, experiencing a severe emotional crisis during the study, and getting pregnant during the study.

### Roles and responsibilities

This study will be conducted in Shahid Beheshti Hospital, Kashan. The trial will be run on an individual basis by the principal investigators. The study will be overseen by the scientific committee every week or when the need arises, in case of any adverse events. Kashan University of Medical Sciences will oversee the overall conduct of the trial. The study team will meet and discuss the problems in the operation of the study every week. DMC will be not needed for the study as the study will involve a small group of patients and will be in a single center.

### Data management and confidentiality

Data will be stored in SPSS software and kept in local storage as well as in cloud storage. Although patients will have to provide their full names and contact details on the consent form, proper measures will be taken to protect their confidentiality. Their names will be replaced with a unique number that will be pasted onto their consent forms and their completed survey questionnaires. Results will be reported in aggregate. The data will not contain any personal information of the participants. Although every effort will be made to keep study records private, in some cases, patient information in this research study can be reviewed by representatives of the University for purposes such as quality control or safety.

## Assessment of variables

### Sleep quality and sleep disturbances

The PSQI evaluates self-reported sleep disturbances and quality in the previous 4 weeks. PSQI measures 7 components of sleep and has 19 items: subjective sleep quality, sleep duration, sleep latency, use of sleeping medication, sleep disturbance, daytime dysfunction, and habitual sleep efficiency [20]. These items are rated in terms of the severity of the problem or frequency on a 4-point Likert scale (not during the past month: 0, less than once a week: 1, once or twice a week: 2, three or more times a week: 3). Each component score has a range of 0 to 3, with greater scores indicating lower sleep quality. The component scores sum yields a PSQI global score which ranges from 0 to 21. Higher scores express worse sleep quality. Statistical analyses also support looking at three factors, which include perceived sleep quality (using sleep latency, subjective sleep quality, and sleep medication variables), sleep efficiency (using sleep efficiency and sleep duration variables), and daily disturbances (using daytime dysfunctions and sleep disturbances variables). The PSQI has the same problems as other self-report inventories in that scores can be minimized or exaggerated by the person completing them [20].

### Sleepiness

The ESS measures daytime sleepiness. It is a self-reported questionnaire where individuals answer questions based on how likely they are to doze off or fall asleep during sedentary activities. It is an attractive alternative to objective methods of assessing sleepiness. ESS has eight items and includes a respondent format “would never doze” = 0, “slight chance of dozing” = 1, “moderate chance of dozing” = 2, and “high chance of dozing” = 3. A total score is calculated by summing the scores of the eight items, ranging from 1 to 24 points with higher values reflecting a higher level of sleepiness. The change score is calculated as the change between the total ESS score at baseline and follow-up [21].

### Insomnia

The Insomnia Severity Index has seven items that evaluate the severity of sleep-onset (initial), early morning awakening (terminal) problems, sleep maintenance (middle), satisfaction with current sleep pattern, noticeability of impairment attributed to the sleep problem, interference with daily functioning, and level of distress caused by the sleep problem. Each item is rated on a 0–4 scale (0 indicating no problem and 4 indicating a very severe problem) with a total score ranging from 0 to 28. The Insomnia Severity Index total scores are categorized as 0–7: not clinically significant insomnia; 8–14: subthreshold insomnia; 15–21: moderate insomnia; and 22–28: severe insomnia [22].

### Physical activity scale

The questionnaire of physical activity based on the metabolic equivalents (MET) will be used to measure physical activity in this study, which includes 9 levels of activity from sleep and rest (METs = 0.9) to intense activity (more than 6 METs) [23].

### Anthropometric indices

A stadiometer (accuracy of 0.5 cm) will be used to measure the Height. Individuals’ weight, with a light dress and without shoes, will be measured by using the scale (accuracy of 0.1 kg). BMI will be calculated as weight (kg) divided by height squared (m^2^). The waist-to-hip ratio will be calculated as the waist circumference divided by the hip circumference. The waist circumference will be measured at the midpoint between the iliac crest and lowest rib, by using a non-stretchable measuring tape during normal expiration [24]. The hip circumference will be horizontally measured at the widest portion of the buttocks [24]. Other anthropometric indices such as extracellular water ratio, visceral fat area, percent body fat, fat-free mass, body fat mass, soft lean mass, skeletal muscle mass, and total body water will be evaluated by Inbody770 model BPM040S12FXX. All anthropometric indices will be measured at the beginning and end of the study.

### Statistical assessment

To investigate if the data follows a normal distribution, we will use the Kolmogorov-Smirnov test. To compare qualitative data between the two groups, a chi-square test will be used. An independent t-test will be used to determine differences in quantitative data between groups. In non-parametric conditions and parametric conditions, the Wilcoxon test and paired *t*-test, respectively, will be used to compare the mean of quantitative variables within the group before and after the intervention. To compare the mean between the two groups, the Mann-Whitney test and *t*-test for non-parametric conditions and parametric conditions, respectively, will be used. Analysis of covariance will be utilized to determine the difference in data changes between the 2 groups at the end of the study. The adjusted *p-*values corresponding to each statistical test will be used to investigate the intervention effects by using the Bonferroni correction to analyze multiple outcomes. Data analysis will be done with SPSS software. We plan to conduct two subgroup analyses, both with strong biological rationale and possible interaction effects. Multiple regression model and analysis of covariance (ANCOVA) will be used to evaluate whether specific variables are confounders for the treatment effect and whether the treatment effect interacts with specific covariates.

We propose to use two analysis sets; the intention-to-treat set, considering all patients as randomized regardless of whether they received the randomized treatment, and the “per protocol” analysis set. Given our expectation that very few patients will crossover or be lost to follow-up, these analyses should agree very closely. We will report reasons for withdrawal for each randomization group and compare the reasons qualitatively. The effect that any missing data might have on results will be assessed via sensitivity analysis of augmented data sets. Dropouts (essentially, participants who withdraw consent for continued follow-up) will be included in the analysis by regression imputation method for missing data. This will be accomplished using a set of repeated imputations created by predictive models based on the majority of participants with complete data. After the imputations are completed, all of the data (complete and imputed) will be combined and the analysis performed for each imputed-and-completed dataset. Rubin’s method of multiple (i.e., repeated) imputation will be used to estimate the treatment effect. No interim analyses will be performed.

### Harms

Adverse events if any, which are directly related to the study intervention, will be monitored and assessed by researchers involved in the study. The patient will be on continuous monitoring. Any such adverse events that arise during the study will be reported to Kashan University of Medical Sciences and the Ethics Committee of Kashan University of Medical Sciences. Any such events will be managed by the treating physician and the cost for the same will be covered under the trial fund.

### Frequency and plans for auditing trial conduct

The study team will meet every week and will conduct at least one on-site visit every week to check the quality of the data, database operation, subject material, and informed consent. This team, totally independent from the authors and competing interests, will have the full right to make the final decision on the trial status.

## Discussion

Given that one of the major causes of sleep disorders is obesity, weight-loss approaches can affect sleep quality. In recent decades, the fasting diet has emerged as a popular option for weight loss. Human trials that investigate the effect of intermittent fasting on sleep quality and sleep disturbances are limited. Also, studies that have investigated the effect of intermittent fasting and daily calorie restriction on anthropometric indices have not reached definitive results. Hence, our study aims to investigate the effect of 8 weeks of intermittent fasting intervention compared with daily calorie restriction on sleep quality and anthropometric indices. The information gained will enhance our understanding of fasting interventions, which can be used to improve clinical dietary recommendations.

### Protocol amendments

Any change in the conductance of the study made in the protocol will be informed in the specified format to Kashan University of Medical Sciences and the IRCT. As the study has followed up, the patients who will be present in the study will be informed about the protocol changes.

## Trial status

Recruitment for the trial starts in September 2022 and is expected to continue recruitment until late December. This is protocol version 1, July 29, 2022.

## Data Availability

The datasets used and analyzed during the study will be available from the corresponding author upon request.

## Appendix

Consent materials: A sample consent form is provided as an appendix to this manuscript. Biological specimens: Laboratory evaluation and storage of biological specimens are not applicable in the current trial.

## Abbreviations

BMI: Body mass index
PSQI: Pittsburgh Sleep Quality Index
FFM: Fat-free mass
MET: Metabolic equivalents
